# Collagenolytic *Enterococcus faecalis* induces DDR1 signaling, proliferation and altered immune infiltrate in colorectal peritoneal metastases

**DOI:** 10.1016/j.sopen.2025.10.011

**Published:** 2025-11-08

**Authors:** Richard Jacobson, Sean Dineen, John Mullinax, Ryan Martin, Sidharth Mishra, Michelle Maurin, Ramani Soundararajan, Timothy Nywening, Andreas Karachristos, Hariom Yadav, Timothy Yeatman, Jason Fleming

**Affiliations:** a Tampa General Hospital Cancer Institute, Tampa, FL, United States of America; b University of South Florida Department of Surgery, Tampa, FL, United States of America; c H. Lee Moffitt Cancer & Research Institute Department of Gastrointestinal Oncology, Tampa, FL, United States of America; d H. Lee Moffitt Cancer & Research Institute Department of Sarcoma, Tampa, FL, United States of America; e University of South Florida Microbiomes Institute, Tampa, FL, United States of America; f University of Texas Southwestern Medical Center Department of Surgery, Dallas, TX, United States of America

**Keywords:** Metastatic colorectal cancer, Intratumoral bacteria, *Enterococcus faecalis*

## Abstract

**Background:**

Intratumoral pathogens are an emerging paradigm in metastatic colorectal cancer (CRC). Overgrowth of *Enterococcus faecalis* was shown to promote local recurrence in the colon, in a fashion dependent on collagenolytic virulence factors. The role of intratumoral enterococci in metastatic CRC is presently unknown.

**Methods:**

We screened resected human metastatic CRC from the liver, lungs, and peritoneal surface for intratumoral bacteria with 16 s rRNA sequencing. We probed the effects of *E. faecalis* on CRC biology in vitro*,* with a focus on collagenolysis and the putative receptor for cleaved collagen, discoidin domain receptor 1 (DDR1) in CT26 CRC cells. We used a syngeneic, orthotopic mouse model of colorectal peritoneal metastases to measure the impact of *E. faecalis* on tumor bulk and immune infiltrate.

**Results:**

Resected metastatic CRC from 70 patients were screened for intratumoral bacteria. *Enterococcus* species were identified in 10/13 patients with CRC peritoneal metastases and were enriched in peritoneal compared to non-peritoneal metastases. *E. faecalis* and CRC cells demonstrated cooperative collagenolysis in a fashion dependent on the secreted virulence factors GelE and SprE*.* Bacterial-induced collagenolysis led to increased DDR1 phosphorylation and downstream effects, specifically proliferation and endocytosis of cleaved collagen. In the mouse model, cell counts indicate intratumoral *E. faecalis* altered the immune compartment of the tumor microenvironment.

**Discussion:**

Collagenolytic *E. faecalis* induce DDR1 pathway activation in CRC cells, alter the immune landscape in mouse models, and are enriched in human CRC peritoneal metastases. Further work is required to determine whether eradication of intratumoral bacteria can change tumor biology.

## Introduction

Colorectal cancer (CRC) develops in direct exposure to the gut microbiota and recent work suggested that intratumoral microbes have a substantial impact on tumor biology [1,2]. CRC metastasizes through either hematogenous or transcolonic/transperitoneal mechanisms that are poorly understood [3]. Transcolonic/transperitoneal spread requires collagen degradation in the tumor microenvironment and leads to colorectal peritoneal metastases (CPM), which are resistant to current therapeutics and have a worse prognosis than sites of hematogenous spread such as the liver or lungs [4,5]. The contribution of collagenolytic intratumoral microbes in the progression of CPM is presently unknown.

*Enterococcus faecalis* is a commensal gram-positive diplococcus capable of rapid expansion of its population, and conversion to a virulent phenotype during periods of metabolic stress such as surgery [6]. The Fsr quorum-sensing system in *E. faecalis* induces expression of two proteases, gelatinase (GelE) and serine protease E (SprE) when population density rises [7]. These virulence factors directly cleave collagen and activate host matrix-metalloproteases, leading to tissue destructive virulence [8,9]. In addition to infectious complications, *E. faecalis* induced a migratory/invasive phenotype in CRC cells in vitro [10]. It led to local recurrence of CRC in mouse models [11]. Notably, the ability of *E. faecalis* to alter in vitro and in vivo tumor biology depended on the expression of collagenolytic virulence factors. The relative abundance of *E. faecalis* in CPM, and its impact on the biology of peritoneal metastases are unknown to date.

Our hypothesis is that collagenolytic intratumoral *E. faecalis* contributes to progression of CPM. The discoidin domain receptor 1 (DDR1) is a putative receptor for cleaved collagen expressed on the surface of CRC cells [12]. High expression is associated with impaired antitumor immunity and poor overall survival in human CRC [13,14]. In this study we measured the contribution of *E. faecalis* to collagenolysis by CRC cells, and downstream activation of the DDR1 pathway in vitro. We used a syngeneic, orthotopic mouse model of CPM to measure the impact of intratumoral *E. faecalis* in vivo. To assess the clinical relevance of *E. faecalis* in CPM we screened 70 metastatic CRC tumors resected from humans for intratumoral bacteria using 16 s rRNA sequencing.

## Materials and methods

### Human tissue collection

Tissue analyzed in this study was collected between 2006 and 2010 as part of the Total Cancer Care Consortium, a prospective biobank that included 18 institutions at the time. All subjects aged 18 or older undergoing resection for primary or metastatic colon or rectal adenocarcinoma were eligible to participate. Receipt of preoperative systemic therapy had no bearing on eligibility. All tumors were collected in a sterile fashion and snap frozen in liquid nitrogen within 20 min of collection. Tumor viability was assessed by a gastrointestinal pathologist on matched hematoxylin and eosin stains of separate paraffin-embedded samples derived from the same tumors as frozen specimens.

### 16 s rRNA analysis of human tissue

Approximately 30 mg of each previously frozen sample (retrieved from −80 °C) was used for bacterial genomic DNA extraction utilizing the QIAamp DNA Microbiome Kit (Qiagen, CA, USA). The purified genomic DNA was then processed for 16S rRNA gene-based metagenomic analysis, specifically targeting the V4 hypervariable region. In brief, the V4 hypervariable region of the bacterial 16S rRNA gene was PCR-amplified using the universal primer set 515F and 806R [15]. Sample-specific barcodes were incorporated during amplification, enabling multiplex sequencing. PCR products were purified using AMPure® XP magnetic beads (Agencourt, Beckman Coulter, CA, USA), and DNA concentrations were quantified with the Qubit dsDNA HS Assay Kit on a Qubit 3.0 fluorometer (Life Technologies, Carlsbad, CA, USA). Equimolar concentrations of purified amplicons were pooled to construct the sequencing library, which was adjusted to a final concentration of 8 pM. Sequencing was performed on the Illumina MiSeq platform using the MiSeq Reagent Kit v3, following the manufacturer's protocol. Raw reads were de-multiplexed, quality-filtered, and clustered using the QIIME pipeline, followed by downstream analysis as previously described [[16], [17], [18], [19]].

### Materials

CT26 CRC cells were used in all experiments and cultured in RPMI-1640 media supplemented with 10 % fetal bovine serum, 100 U/mL of penicillin G, and 100 μg/mL streptomycin. *E. faecalis* strain V583 and ΔgelEΔsprE were obtained from Dr. Benjamin Shogan (University of Chicago, USA) and were plated on tryptic soy agar, then grown overnight in tryptone yeast liquid media. Balb/c mice were purchased from Charles River Laboratories.

### Collagen degradation assays

Were performed per the manufacturer's instructions as previously described [8]. CT26 cells at the indicated plating density were grown overnight in RPMI. To create supernatant, bacterial cultures in tryptone yeast were normalized to an optical density of 0.1 at 600 nm, then passed through a 0.2 μm filter and added to the reaction plate at the indicated dilutions. 2 μL DQ type I and IV collagen (Thermo Fisher, USA) diluted 5:1 in sterile water were added just prior to introduction into the plate reader. The total reaction volume was 200 μL. Change in fluorescence over time (relative fluorescence units per second, RFU/s) at 480/520 nm was determined kinetically over the initial 30 min of the reaction, when it demonstrated pseudo-first order kinetics. All experiments were run in triplicate.

### MTT assay

5000 CT26 cells per well were plated on a 96 well plate and grown overnight in 100 μL RPMI. Bacterial supernatant from liquid cultures of *E. faecalis* V583 in tryptone yeast was harvested as above. 100 μL of supernatant or sterile filtered tryptone yeast media as a vehicle control were added to each well. At each timepoint, 10 μL MTT reagent (3-(4,5-dimethylthiaxol-2-yl)-,5 diphenyltetrazolium bromide, ThermoFisher) was added to each well and incubated for 3 h. After incubation, 150 μL media was removed and 50 μL DMSO was added and mixed. The plate was incubated for 10 min and final absorbance was read at 570 nM.

### Western blot

CT26 cells were cultured to 80 % confluency then treated with bacterial supernatant or filtered bacterial media as a negative control for the times indicated. Cells were then lysed in RIPA buffer supplemented with protease and phosphatase inhibitors. Lysates were centrifuged at 12,000 ×*g* for 15 min at 4 °C. The supernatant was collected, and protein was quantified. A 10 % SDS-polyacrylamide gel was loaded with 20 μg protein per lane, and electrophoresis was run at 100 V until clear band separation was observed. Proteins were transferred to a PVDF membrane at 25 V over 30 min. Membranes were blocked with 5 % non-fat dry milk for an hour, and then stained with anti-DDR1, anti-phospho-DDR1 (Y796), and anti-beta actin antibodies (R&D systems, MN, USA) overnight, followed by HRP-conjugated secondary antibodies for 1 h at room temperature.

### Collagen endocytosis assay

CT26 cells were grown for 24 h on chamber slides in RPMI, then incubated with V583 supernatant or sterile media overnight as described above. DQ-type I collagen (Thermo Fisher, USA) was added to the incubation at various timepoints prior to imaging, as indicated. Chambers were washed x3 with PBS, then fixed in 4 % formaldehyde. A Hoechst counterstain was applied, and cells were imaged on a Leica SP8 confocal microscope with a 100× oil immersion objective, at an excitation wavelength of 488 nm and emission detection using a bandpass filter set to 500-550 nm.

### Mouse model of colorectal peritoneal metastases

We utilized 8-week-old Balb/c mice (Charles River) and provided standard chow diet and tap water *ad libidum*. Mice received 40 mg/kg subcutaneous cefoxitin the day of surgery. General inhalational anesthesia was induced with isoflurane. Each mouse had a midline laparotomy followed by a colotomy and repair with interrupted nylon suture to stimulate a predictable site of tumor development. A leak test was performed with a saline enema to ensure patency and integrity of the repair. Next, 5*10^4^ CT26 cells in RPMI were instilled into the open abdominal cavity using aseptic technique. The CT26 cells had been coincubated for 24 h with either 1) sterile bacterial media as a vehicle control, 2) *E. faecalis* V583, or 3) *E. faecalis ΔgelEΔsprE* at a multiplicity of infection of 100 prior to administration. All mice were monitored for signs of intraabdominal sepsis and following recovery, again provided diet *ad libidum*. At postoperative day 21 mice were sacrificed, and intraabdominal tumor was quantified using a peritoneal carcinoma index score as previously described and collected for further analysis [20].

### Immune fluorescence panel

Tumors harvested from the rectosigmoid junction at the site of the anastomotic injury in the mouse model were fixed in formalin and embedded in paraffin. Samples were stained with the OPAL 7 Color Immunohistochemistry Kit (PerkinElmer, Waltham, MA) with primary antibodies against CD4, CD8, FoxP3 and F/480 (Abcam). Deparaffinization, antigen retrieval, and staining, were performed using the OPAL manufacturer's protocol. DAPI counterstaining was applied to all slides. We generated multi-layer TIFF images and loaded them into HALO 121 for cell counts. We set a positivity threshold for each marker, and the entire image was analyzed including the colon and pericolonic tumor. Cell counts are reported as positive cells/ mm^2^. Between-group comparisons were performed using unpaired *t*-tests.

### Statistical analyses

Ordinary one-way ANOVA was utilized to compare means between three or more independent groups. t-tests were used to compare means between two groups.

### Ethics approval

All animal experiments were approved by the Institutional Animal Care and Use Committee at the University of South Florida. All tissue collection performed as part of the Total Cancer Care consortium was approved by Institutional Review Boards at each participating site. Locally, this was performed at the University of South Florda.

## Results

### Human CRC peritoneal metastases are enriched with enterococci compared to hematologic metastases

We hypothesized that CPM are enriched with collagenolytic bacteria such as enterococci because transcolonic/transperitoneal spread across multiple collagen layers is required for development of peritoneal metastases. To test this hypothesis, we analyzed a cohort of resected metastatic colorectal adenocarcinoma from 70 distinct patients.

**Fig. 1:**
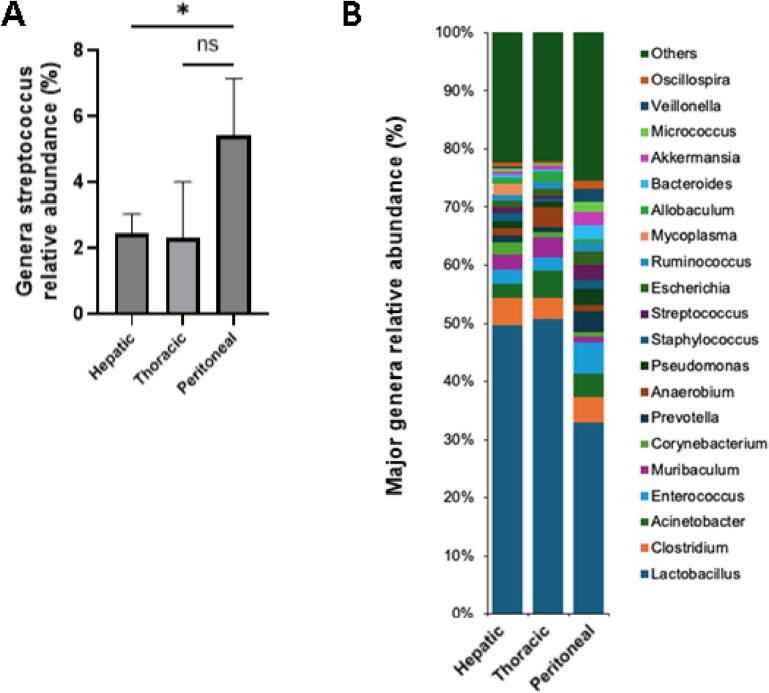
Human CRC peritoneal metastases are enriched with *Enterococcus* species. A) Mean relative abundance of genus *Enterococcus* in 13 peritoneal and 10 thoracic and 45 hepatic metastases resected from human subjects. Enterococcal DNA was recovered in peritoneal metastases from 10/13 subjects (76.9 %). It was recovered in thoracic tumors from 4/10 subjects (40.0 %) and in hepatic tumors from 26/45 subjects (57.8 %). B) Relative mean abundance of all major genera detected in resected metastases from all sites, illustrating a distinct population in peritoneal metastases compared to other sites *p < 0.05 one-way ANOVA.

We analyzed 70 resected specimens (45 hepatic, 10 thoracic, 13 peritoneal, 2 cerebral) with 16 rRNA sequencing. Tumor cell viability was similar between groups (86 % peritoneal, 89.5 % thoracic, 83.4 % hepatic, *p* > 0.48 all comparisons, one-way ANOVA). The prevalence of *Enterococcus* in CPM was previously unknown. *Enterococcus* species were identified in peritoneal metastases from 10/13 subjects (76.9 %). They were identified in thoracic tumors from 4/10 subjects (40.0 %) and in hepatic tumors from 26/45 subjects (57.8 %). The mean relative abundance of *Enterococcus* species was significantly higher in CPM than in hepatic metastases (5.44 % vs 2.44 % *p* = 0.04 one-way ANOVA) and approached significance vs thoracic metastases (5.44 % vs 2.30 % *p* = 0.11 one-way ANOVA, Fig. 1A). Cerebral metastases were excluded from this analysis due to low sample size. When tumors were grouped by route of metastatic spread (CPM via the transperitoneal route, all others via the hematogenous route), we observed *Enterococcus* species were enriched in CPM compared to hematologic metastases (5.44 % vs 2.41 %, *p* = 0.04 student's *t*-test). Fig. 1B illustrates the mean relative abundance of all major bacterial genera identified in peritoneal, hepatic and thoracic metastases. We observed a relative enrichment in collagenolytic genera such as *Enterococcus, Streptococcus* and *Pseudomonas* and a relative paucity of non-collagenolytic *Lacotbacillus* in CPM.

### *E. faecalis* increases collagenolysis by CT26 CRC cells via the secreted virulence factors GelE and SprE

**Fig. 2:**
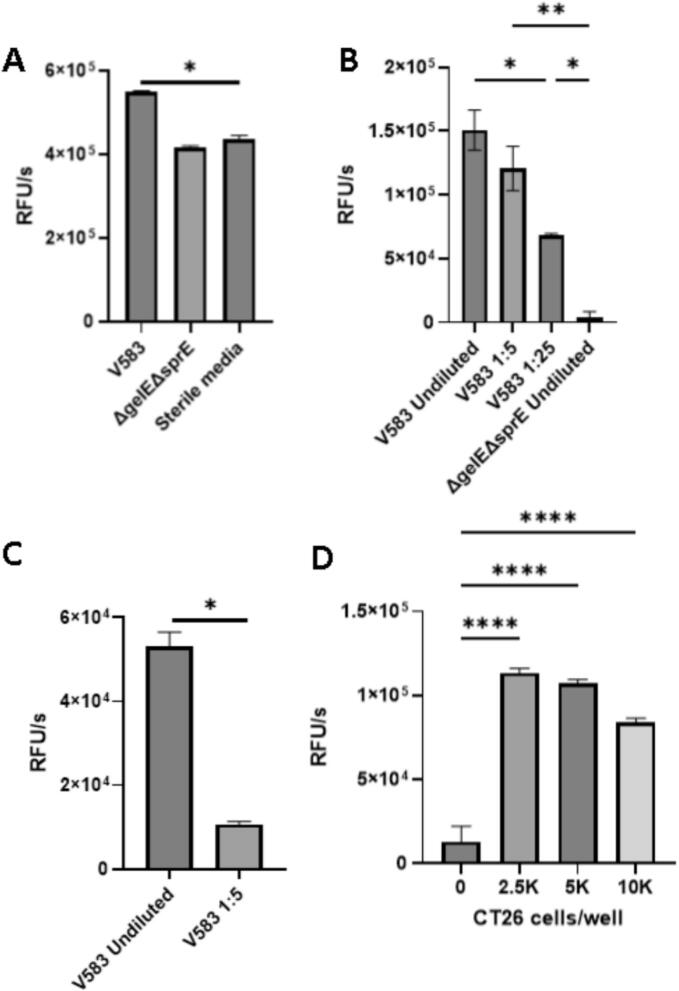
*E. faecalis* and colorectal cancer cells cooperatively degrade types I and IV collagen in a fashion dependent on bacterial expression of secreted GelE and SprE. A) Type I collagen degradation assay of supernatant after 24-h coculture with CT26 cells demonstrates increased collagenolysis in the presence of bacteria, only when GelE and SprE are expressed. B) Type I collagen degradation assay of CT26 cells grown to confluence with serial dilutions of V583 supernatant and undiluted ΔGelEΔSprE as a negative control. C) Type IV collagen degradation assay of CT26 cells grown to confluence undiluted vs diluted V583 supernatant, again suggesting concentration-dependent collagenolysis. D) Type I collagen degradation assay with constant concentrations of bacterial supernatant and increasing seeding density of CT26 cells demonstrates that both bacterial and cancer cell factors contribute to collagenolysis, and an inverse relationship between CT26 seeding density and collagenolysis RFU/s: relative fluorescence units per second. Data shown are reaction velocities calculated by change in fluorescence over time during the initial 30 min of each reaction when they displayed pseudo first order kinetics. All experiments were run in triplicate. * *p* < 0.05 one way ANOVA.

*E. faecalis* has been shown to increase proliferation of CRC cells via multiple mechanisms, but its impact on metastogenesis remains unclear [21,22]. As transcolonic/transperitoneal spread requires degradation of submucosal and peritoneal surface collagen, we measured the effect of *E. faecalis* on collagen degradation by CT26 cells. *E. faecalis* express collagenolytic virulence factors, and CT26 cells express pro-matrix metalloproteases and their inhibitors, but the effect of bacteria on net collagenolysis by CRC cells is unknown. We first incubated CT26 cells overnight with *E. faecalis* strain V583 or its non-collagenolytic derivative mutant ΔgelEΔsprE and measured type I collagenolysis in the supernatant of this coculture. We observed increased collagenolysis only in the presence of parent strain, compared to ΔgelEΔsprE and vehicle controls (549.2 ± 2.0 RFU/s*10^3^ V583; 415.8 ± 3.0 *ΔgelEΔsprE*; 436.6 ± 5.2 vehicle) (Fig. 2A, * indicates *p* < 0.05 one-way ANOVA). We next wanted to measure the impact of bacterial supernatant on collagenolysis by live CT26 cells, to confirm that bacterial secreted factors contributed to collagenolysis. We observed a dose response in type I collagenolysis when serial dilutions of V583 supernatant were added to live CT26 cells, but no response to *ΔgelEΔsprE* supernatant (150.7 ± 15.6 RFU/s*10^3^ V583 undiluted; 120.7 ± 17.4 diluted 1:5; 68.3 ± 1.2 diluted 1:25; 3.5 ± 5.1 *ΔgelEΔsprE* undiluted) (Fig. 2B). The peritoneal surface contains types I, and IV collagen, both of which are known to be degraded by *E. faecalis*. To generalize our findings to type IV collagen, we performed a type IV collagen degradation assay. A similar concentration-dependence was observed when serial dilutions of V583 supernatant were added to live CT26 cells and type IV collagenolysis was measured (53.0 ± 3.5 RFU/s*10^3^ V583 undiluted; 10.7 ± 0.6 diluted 1:5) (Fig. 2C) To assess whether CRC cells contributed to the reaction or if it were driven entirely by bacteria, we plated serially increased densities of live CT26 cells in the presence of constant concentrations of V583 supernatant and observed an increase in collagenolysis when CRC cells were added to the reaction (129.1 ± 6.5 RFU/s*10^3^ without CT26; 113.5 ± 1.5 with 2.5 K cells/well), but a slight inverse relationship between CT26 seeding density and total collagenolysis (Fig. 2D).

### Collagenolysis by *E. faecalis* and CRC cells induces DDR1 phosphorylation and its downstream effects

**Fig. 3:**
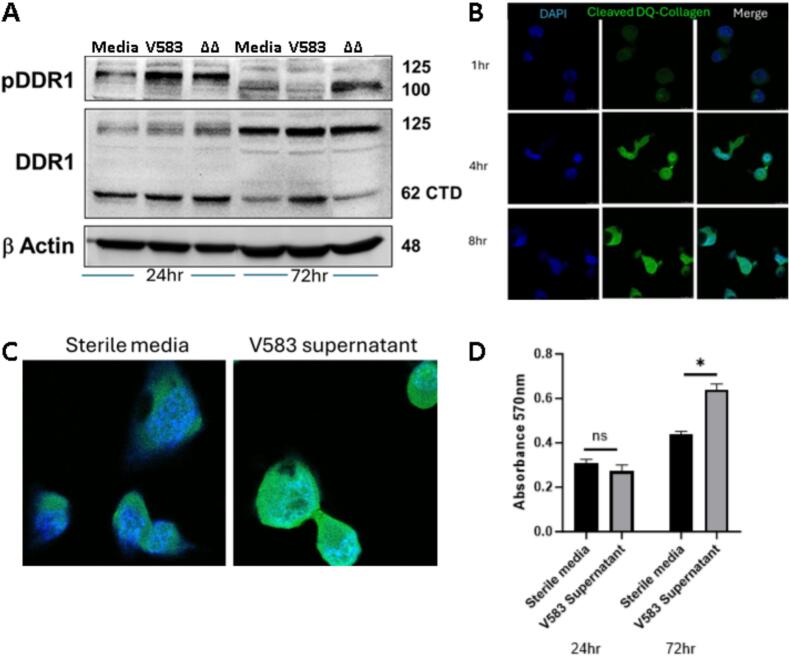
DDR1 phosphorylation and downstream effects are increased in CT26 cells the presence of collagenolytic *E. faecalis.* A) Western blot of CT26 cellular lysate after 24- and 72-h incubation with V583 *and ΔgelEΔsprE* supernatants demonstrates increased DDR1 phosphorylation at 24 h, and increased detection of the 62kD cleaved C-terminal domain, a late marker of pathway activation, at 72 h with exposure to V583 supernatant. B) Fluorescence cytometry of sterile CT26 cells after incubation with V583 supernatant and dye-quenched type I collagen demonstrating cytoplasmic staining of cleaved collagen increasing with time. C) Cytoplasmic staining of cleaved collagen was increased in the presence of V583 supernatant compared to sterile controls after an 8-h incubation. D) MTT assay demonstrating increased proliferation of CT26 cells after 72 but not 24-h incubation with V583 supernatant compared to sterile controls.

The discoidin domain receptor 1 (DDR1) is activated in response to collagen degradation in other cancer sites such as pancreatic ductal adenocarcinoma, inducing aggressive biology in the presence of cleaved collagen [23]. Its expression is a poor prognostic factor in CRC, however the impact of bacterial and intratumoral collagenolysis on DDR1 pathway activation is unknown [24]. We performed a western blot of cellular lysate after incubation of CT26 cells with V583 supernatant, *ΔgelEΔsprE* supernatant, or sterile media as a vehicle control. We observed increased staining of the 125kD phospho-DDR1 band after 24 h incubation with V583 supernatant compared to *ΔgelEΔsprE* supernatant and vehicle. After 72 h incubation, we observed no band at 125kD, but rather a fragment at a lower molecular weight. We did observe increased staining of the 62kD cleaved C-terminal domain of DDR1, a late signal of pathway activation [25] (Fig. 3A). Raw images of the complete gel are shown in Fig. S1. In pancreatic ductal adenocarcinoma, DDR1 activation by cleaved collagen leads to endocytosis and further catabolism of cleaved collagen [26]. We designed an assay to test this effect in CRC cells, wherein CT26 cells were incubated over a time course with dye-quenched type I collagen that fluoresces only when cleaved. We performed fluorescence cytometry that demonstrated progressively increased cytoplasmic staining of Fluorescein Isothiocyanate (FITC)-tagged cleaved collagen at each sequential timepoint (Fig. 3B), indicating endocytosis of cleaved collagen. We observed that V583 supernatant increased FITC staining in the cytoplasm compared to sterile controls (Fig. 3C). Cellular proliferation is a downstream effect of DDR1 signaling in multiple cancers [27]. We performed an MTT assay of CT26 cells incubated with V583 supernatant versus sterile vehicle control and observed increased proliferation at 72, but not 24 h compared to vehicle based on absorbance at 570 nm (0.61 ± 0.2 V583 vs 0.42 ± 0.2 vehicle, *p* < 0.05 unpaired *t-*test) (Fig. 3D).

### Intratumoral *E. faecalis* alters the immune microenvironment in a mouse model of CRC peritoneal metastases

**Fig. 4:**
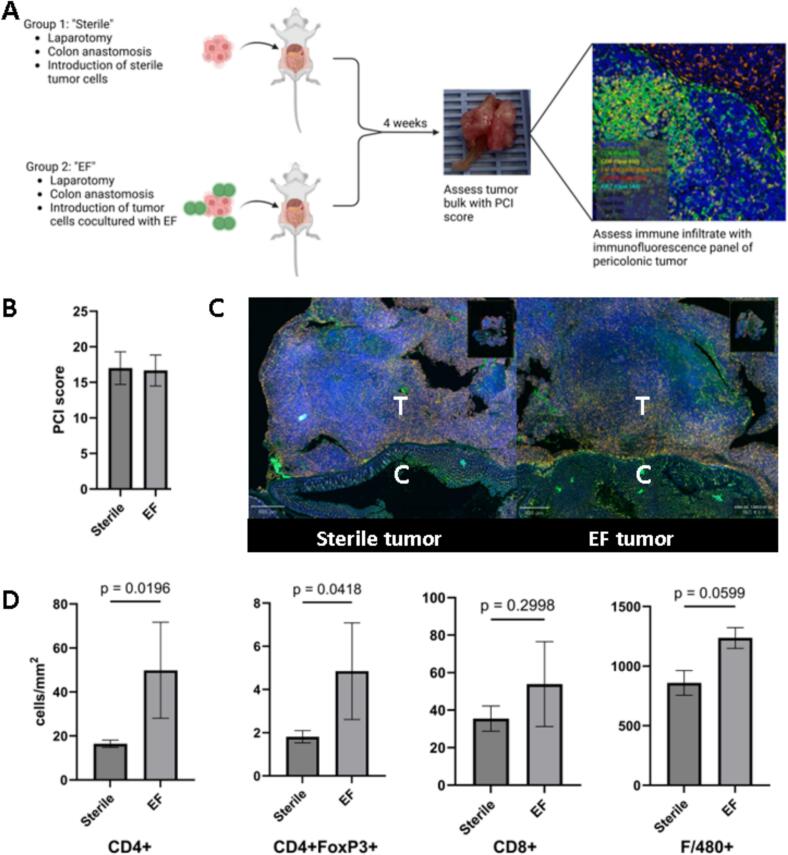
Presence of intratumoral *Enterococcus faecalis* is associated with altered immune infiltrate in the tumor microenvironment in a mouse model. A) Schematic of the mouse model and example immunofluorescence panel of normal murine colonic mucosa at high power - Blue: DAPI, Green: CD4+, Red: FoxP3+. Yellow: CD8+, Orange: F/480+. B) Tumor bulk as measured by peritoneal carcinomatosis index calculated on necropsy was not different between groups. C) Low power immune fluorescence image of perianastomotic colon and associated sterile tumor demonstrating dense infiltrate of macrophages and lymphocytes at the periphery of the tumor with less infiltrate in the tumor core. Low power image of tumor seeded with *E. faecalis* strain V583 demonstrated a similar overall pattern. D) Cell counts with increased density of CD4+ and CD4 + FoxP3+ in tumors seeded with *E. faecalis*, and a trend toward increased F/480+ cells lymphocytes compared to sterile tumors. Images are representative of *n* = 4 mice per treatment group. EF: tumors seeded with *E. faecalis*. Sterile: tumors seeded with sterile bacterial media as a vehicle control. (For interpretation of the references to color in this figure legend, the reader is referred to the web version of this article.)

We designed a mouse model to test the hypothesis that collagenolytic *E. faecalis* would cause immune exclusion in CRC peritoneal metastases. Four mice per group underwent laparotomy, rectosigmoid anastomosis and seeding of 5*10^4^ CT26 cells co-incubated with either V583 or vehicle control. They were sacrificed at 3 weeks postoperative. Peritoneal carcinomatosis index (PCI) scores were calculated at the time of sacrifice and rectosigmoid tumors were harvested for analysis with an immune fluorescence panel (schema Fig. 4A, details Fig. S2). PCI was high in all groups, driven by bulky tumor in the pelvis, abdominal wall and back/flank, and did not differ significantly between groups (17.25 sterile vs 17.0 V583) (Fig. 4B). The microenvironment of tumor seeded with V583 demonstrated increased density of CD4+ (49.85 vs 16.46 cells/mm^2^, *p* = 0.02 unpaired *t-*test) and CD4 + FoxP3+ (4.85 vs 1.81 cells/mm^2^, *p* = 0.04) cells in the tumor microenvironment. We also observed a trend toward increased F/480 positive macrophages (1236.0 vs 859.4 cells/mm^2^, *p* = 0.06). The difference in CD8+ cells (53.92 vs 35.49 cells/mm^2^, *p* = 0.30) was not statistically significant (Fig. 4C, D).

## Discussion

We detected *Enterococcus* DNA in the majority of resected human CPM, and found it was relatively enriched in peritoneal compared to hematogenous metastases. We observed supra-additive collagenolysis between *E. faecalis* and CT26 colorectal cancer cells. Bacterial-mediated collagenolysis induced DDR1 phosphorylation and the downstream effects of collagen endocytosis and cellular proliferation in vitro, further elucidating the biologic effect of *E. faecalis* on CRC biology. These findings are novel in the colorectal disease site and suggest collagenolysis-driven DDR1 activation may underlie the prior finding of aggressive tumor biology induced in CRC by *E. faecalis* [10,11]*.*

We expected that DDR1 activation would translate to immune exclusion in the mouse model of CPM, however we observed increased immune infiltrate in tumors seeded with *E. faecalis*. *E. faecalis* induce epithelial inflammation through several pathways that could have contributed to this observation. Further characterization of these immune cells is required to determine whether they represent pro-or anti-tumor infiltrate, however our finding that Treg-like CD4 + FoxP3+ cells were more prevalent in tumors bearing *E. faecalis* indicates that at least a portion of the infiltrate was pro-tumor. We did not observe a difference in tumor bulk in our mouse model between tumors seeded with *E. faecalis* and sterile ones, likely representing a limitation of our model's small sample size and quantification technique. Future studies should include mouse survival as an endpoint, and more precise fluorescent means of quantifying tumor bulk. The observation that *ΔgelEΔsprE* did not survive to endpoint in the tumor microenvironment may indicate that collagenolysis gives bacteria a survival advantage in the tumor microenvironment, however further investigation is needed to test this hypothesis.

## Conclusion

Taken together, these observations align with the hypothesis that collagenolytic *E. faecalis* in the tumor microenvironment contributes to progression of peritoneal metastases. Our work supports the idea that the compartments of the tumor microenvironment (epithelial, stromal, immune, vascular, microbial) are interdependent. Bacteria and epithelial cells degraded stromal collagen that fed back a growth signal to the epithelium and impacted immune infiltrate. Intratumoral bacteria are targetable with existing pharmaceuticals, and future basic and translational work focused on manipulating the intratumoral microbiome is critical to determine whether they are a clinically relevant target.

## CRediT authorship contribution statement

**Richard Jacobson:** Conceptualization, Formal analysis, Funding acquisition, Investigation, Methodology, Resources, Writing – original draft, Writing – review & editing. **Sean Dineen:** Methodology, Resources, Writing. **John Mullinax:** Methodology, Resources, Writing. **Ryan Martin:** Conceptualization, Data curation, Investigation, Methodology. **Sidharth Mishra:** Data curation, Formal analysis, Investigation. **Michelle Maurin:** Data curation, Formal analysis, Investigation. **Ramani Soundararajan:** Data curation, Formal analysis, Investigation. **Timothy Nywening:** Methodology, Resources, Writing – review & editing. **Andreas Karachristos:** Resources, Supervision, Writing – review & editing. **Hariom Yadav:** Conceptualization, Formal analysis, Investigation, Methodology, Writing – review & editing. **Timothy Yeatman:** Methodology, Resources, Writing – review & editing. **Jason Fleming:** Conceptualization, Formal analysis, Methodology, Resources, Writing – review & editing.

## Ethics approval

The enclosed work was approved by the Institutional Review Board and the Institutional Animal Care and Use Committee at the University of South Florida.

## Funding sources

The enclosed work was funded by a grant to RJ from the 10.13039/100008454American Society for Parenteral and Enteral Nutrition Rhoads Research Foundation.

## Declaration of competing interest

Richard Jacobson reports financial support was provided by American Society for Parenteral and Enteral Nutrition Rhoads Research Foundation. If there are other authors, they declare that they have no known competing financial interests or personal relationships that could have appeared to influence the work reported in this paper.

## Appendix A Supplementary data

Supplementary figures Image 1
