# Supplementary material for: Collagenolytic Enterococcus faecalis induces DDR1 signaling, proliferation and altered immune infiltrate in colorectal peritoneal metastases
Source: Surg Open Sci. 2025 Nov 8;28:65–72. doi: 10.1016/j.sopen.2025.10.011 (PMC12664096; doi:10.1016/j.sopen.2025.10.011)
Supplement: Supplementary file 1 — Supplementary figures [file mmc1.docx]

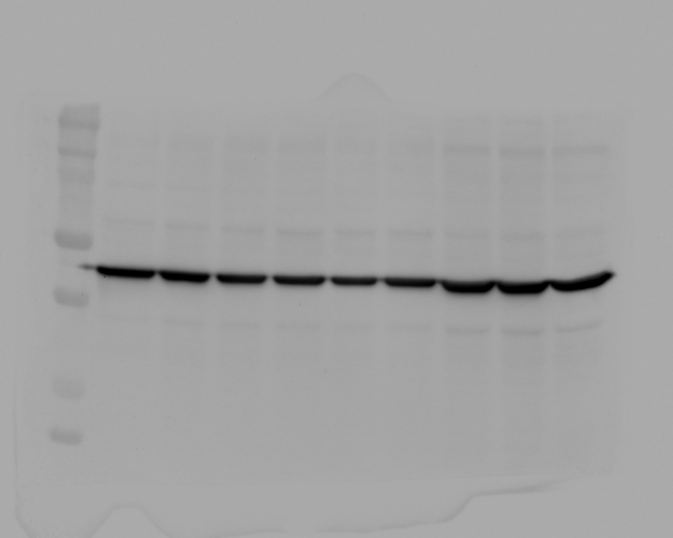
**Actin**


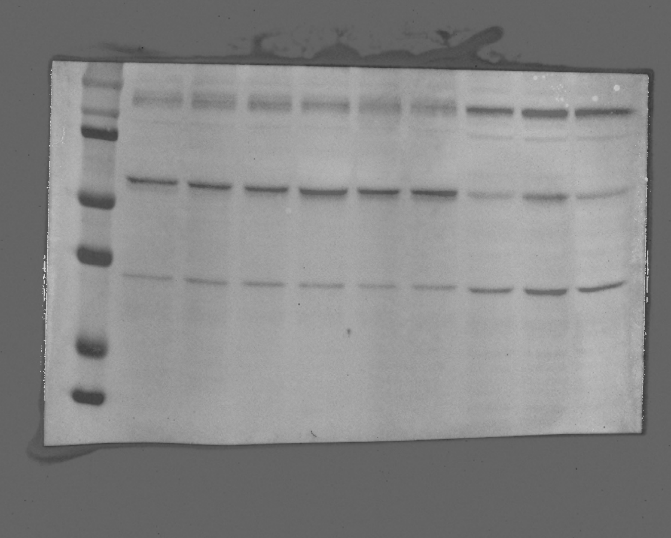
**DDR1**


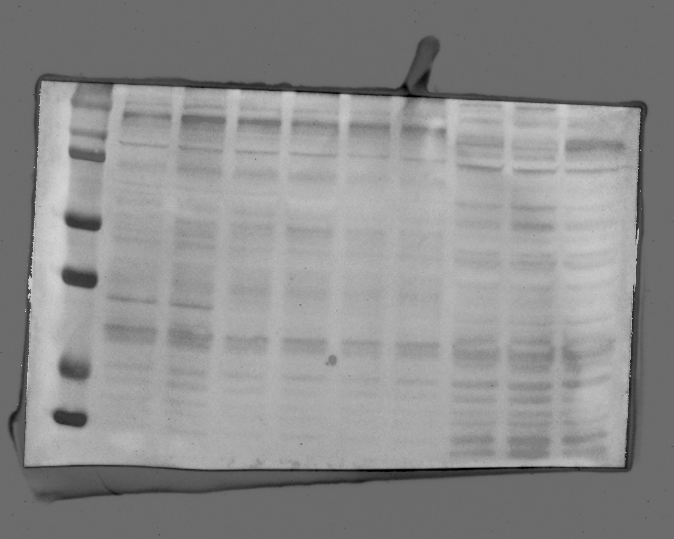
**phospho-DDR1**

**Figure S1:** Raw images of western blot with sequential stains for actin, DDR1, and phosphorylated DDR1. Lanes 1-3 are the 24 hour incubation of V583 supernatant with CT26 cells. Lanes 4-6 are a 24 hour incubation of live V583 with CT26 cells. Lanes 7-9 are a 72 hour incubation of V583 supernatant with CT26 cells.


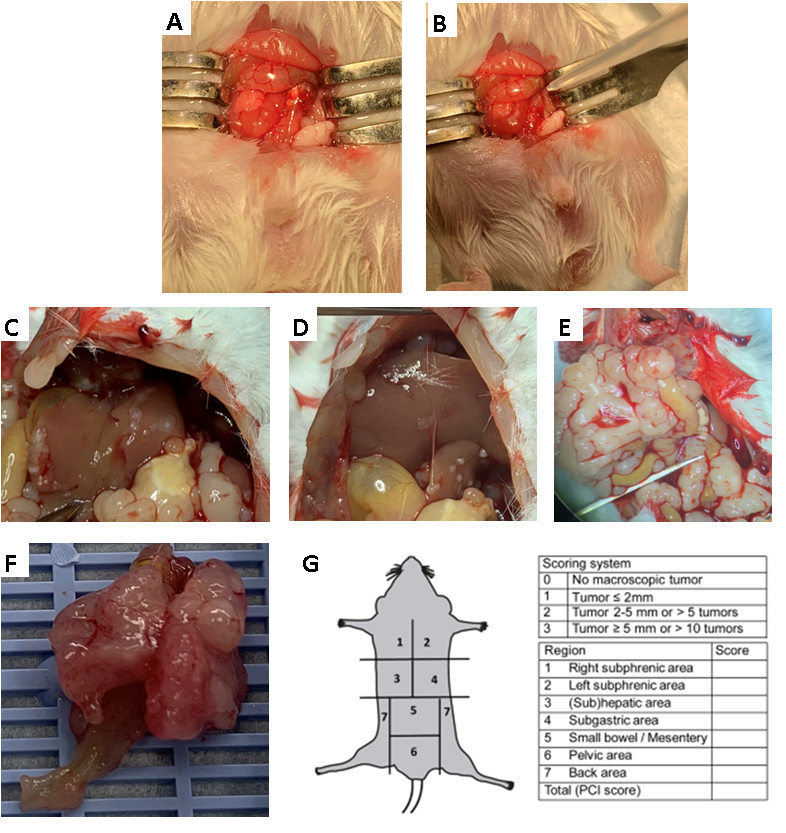


**Figure S2:** Orthotopic mouse model of CPM. A) A laparotomy was made and a small colonic injury made and repaired to induce anastomotic recurrence. B) 50,000 CT26 cells were introduced into the peritoneal cavity via pipette and allowed to distribute physiologically throughout the peritoneal cavity. Cells were either sterile or inoculated with *E. faecalis.* C) Example necropsy photo of the left subphrenic area with peritoneal tumors, scored a 2. D) Subhepatic area with low volume tumor, scored a 1. E) Small bowel mesentery with large volume disease, scored a 3. F) Pelvic/anastomotic recurrence at the site of colonic injury, scored a 3. G) Murine peritoneal carcinoma index used in this study, as validated by Bastianen et al. *Laboratory Investigation,* 2020.
